# Hydroxyethyl Starch-Based Nanoparticles Featured with Redox-Sensitivity and Chemo-Photothermal Therapy for Synergized Tumor Eradication

**DOI:** 10.3390/cancers11020207

**Published:** 2019-02-11

**Authors:** Chan Yu, Chuqi Liu, Shaocong Wang, Zheng Li, Hang Hu, Ying Wan, Xiangliang Yang

**Affiliations:** National Engineering Research Center for Nanomedicine, College of Life Science and Technology, Huazhong University of Science and Technology, Wuhan 430074, China; yuchan1202@hust.edu.cn (C.Y.); liuchuqi@hust.edu.cn (C.L.); MG1830102@smail.nju.edu.cn (S.W.); lizh@hust.edu.cn (Z.L.); hanghu@cczu.edu.cn (H.H.)

**Keywords:** hydroxyethyl starch, nanoparticle, doxorubicin, indocyanine green, redox-sensitivity, cancer chemo-photothermal therapy

## Abstract

Chemo-photothermal combination therapy could achieve synergistically enhanced efficiency against tumors. Nanocarriers with good safety and high efficiency for chemo- photothermal therapy are pressingly needed. A new type of hydroxyethyl starch (HES) based on nanoparticles (NPs) loaded with doxorubicin (DOX) and indocyanine green (ICG) was, thus, developed in this study. DOX-loaded HES conjugates with redox-sensitivity (HES-SS-DOX) were first synthesized and they were then combined with ICG to self-assemble into HES-SS-DOX@ICG NPs with controlled compositions and sizes via collaborative interactions. The optimal HES-SS-DOX@ICG NPs had good physical and photothermal stability in aqueous media and showed high photothermal efficiency in vivo. They were able to fast release the loaded DOX in response to the redox stimulus and the applied laser irradiation. Based on the H22-tumor-bearing mouse model, these NPs were found to tendentiously accumulate inside tumors in comparison to other major organs. The HES-SS-DOX@ICG NPs together with dose-designated laser irradiation were able to fully eradicate tumors with only one injection and one single subsequent laser irradiation on the tumor site during a 14-day treatment period. In addition, they showed almost no impairment to the body. The presently developed HES-SS-DOX@ICG NPs have good in vivo safety and highly efficient anti-tumor capability. These NPs in conjugation with laser irradiation have promising potential for chemo-photothermal cancer therapy in the clinic.

## 1. Introduction

Most chemotherapeutic molecules for cancer treatment show no specificity and they are generally quickly excreted from the body by glomerular filtration or entrapped by the reticulo-endothelial system (RES) once administered intravenously. As a result, they commonly show limited efficiency with low bioavailability while leading to various side effects [1]. Currently, a lot of effort has been made to administer these chemotherapeutic molecules using different nanoscale vehicles with the aim to prolong their circulation in the bloodstream, enhance their therapeutic efficiency, and reduce their systematic toxicity [1,2]. Among different nanocarriers, polymer-conjugates carried with anticancer drugs have attracted more interest in cancer therapy since such conjugates are preferably accumulated in solid tumors and could reduce the toxicity of the loaded drugs [2]. Polymer-conjugates intended for drug-delivery are generally engineered by attaching the drug molecules to water-soluble or hydrophilic polymers through cleavable spacers [2,3], and the employed polymers usually have their molecular weight higher than the limit of the renal threshold, which enables the resulting conjugates to preferentially deliver drugs toward tumors via the prolonged circulation and the enhanced permeability and retention (EPR) effect [4]. To date, a large number of polymer-conjugates bearing anti-tumor drugs have been developed and some of them contain more than a single kind of drug [5]. However, many polymer-conjugates release the loaded drugs by hydrolysis of spacers, which often causes their poor stability while resulting in unwanted side effects on normal cells [6].

Hydroxyethyl starch (HES) is a water-soluble polysaccharide derivative and its properties can be tuned by several major parameters such as molecular weight, degree of hydroxyethyl substitution, and the substitution pattern (C-2/C-6 substitution ratio) alone or in combination [7]. HES has several advantages such as water-solubility, good biocompatibility, α-amylase-mediated degradability, and high parenteral dose tolerance [7,8]. In our previous study, a type of HES-doxorubicin (DOX) conjugate (HES-SS-DOX) was engineered for anti-tumor chemotherapy by using a disulfide-bond bridged spacer rather than hydrolysis-related spacers [9]. Since disulfide bonds in HES-SS-DOX conjugates can be easily cleaved by glutathione (GSH) via the redox reaction while the intracellular GSH level of the tumor cells is known to be several times higher than that in the normal cells [10]. The HES-SS-DOX conjugate should, thus, be able to deliver DOX against tumors via prolonged circulation. Meanwhile, the release of the loaded DOX is rapidly accumulated at tumor sites and/or internalized by tumor cells. The optimal HES-SS-DOX conjugate shows a certain ability to suppress the growth of tumors. It is capable of significantly reducing the toxic side effects associated with free DOX [9]. Nevertheless, somewhat less than that, the therapeutic effect of the HES-SS-DOX conjugate needs to be essentially improved since they are unable to eradicate tumors in the treatment period, which is being administered with multiple injections [9].

Many studies on clinical cancer therapy have revealed that chemotherapy can cause several adverse effects such as the possible stem-cell derivatization of cancer cells, the induced multi-drug resistance, and the tumor metastasis. In addition, chemotherapy alone is frequently inadequate in a large number of cancer therapy cases [11,12,13]. Therefore, many efforts have now been devoted to developing combination cancer therapies, including chemo/photothermal therapy (PTT), chemo/photodynamic therapy (PDT), chemo/immunotherapy, and PTT/PDT, to enhance the therapeutic efficiency while minimizing the side effects of chemotherapeutics [12,13]. Among these combination therapies, the chemo-photothermal therapy based on near infrared (NIR) theranostic agents has attracted increasing clinical attention in recent years. In this mode, the applied photothermal agent is able to absorb NIR light and to convert it into heat-induced cytotoxicity to cancer cells in a noninvasive and harmless manner while working together with the applied chemotherapeutics to achieve synergistically enhanced therapeutic efficacy [14,15].

To date, many small molecules with an imaging nature have been exploited to guide the therapeutic process, track the delivery of drugs, and evaluate the efficacy of cancer treatments [13,15]. Some of them can act as both imaging and photothermal agents [10,13,15,16]. Despite the rapid development of photothermal agents, the small molecules that have a suitable NIR excitation window, meet the in vivo safety requirements, and show high photothermal conversion efficiency for cancer therapy are still few. Indocyanine green (ICG), which is a tri-carbocyanine dye with its maximum absorption and emission at around 740 and 800 nm [15,16], is a type of dual-function agent. In addition to its many clinic applications in diagnosis and drug tracing, ICG has also been used as a photothermal agent together with other chemotherapeutics for cancer chemo-photothermal therapy [17,18,19]. Besides these, ICG can also convert the absorbed NIR light to trigger the generation of reactive oxygen species, which results in photodynamic cytotoxicity to the targeted cancer cells [16,20]. Despite these mentioned advantages, ICG has some shortcomings, including instability in aqueous media, self-aggregation, and a very short half-life in the circulatory system [16,21]. Therefore, ICG needs to be delivered using suitable carriers if it is intended for in vivo use.

In cases of chemo/PTT therapies, it has been suggested that chemotherapy and hyperthermia should be simultaneously performed to achieve the most efficient synergistic effects [17,20,22]. Hence, the chemotherapeutic drug and the photothermal agent have to be synchronously delivered to the tumor cells if they are systemically administered. In recent years, several nanoparticle-based carriers were exploited to deliver DOX and ICG for imaging-guided cancer chemo-photothermal therapy and each treatment was effective to some extent [14,17,20]. Considering the good in vivo biocompatibility but limited efficiency of HES-SS-DOX conjugates, in this study, a new type of HES-SS-DOX@ICG nanoparticles (NPs) was developed for fully eradicating tumors. These HES-SS-DOX@ICG NPs were prepared by directly loading ICG onto the HES-SS-DOX conjugates, basing on the collaborative constraints involving in electrostatic, π–π stacking, and hydrophobic interactions [23]. These HES-SS-DOX@ICG NPs had well-controlled composition and stability while showing high anti-cancer efficacy with good in vivo safety. The optimized HES-SS-DOX@ICG NPs together with NIR laser irradiation were able to fully eradicate tumors with only one single dose injection and the followed single irradiation within a 14-day treatment period. The obtained results demonstrate that these NPs in combination with designated laser irradiation have promising potential in clinical cancer chemo-photothermal therapy.

## 2. Materials and Methods

### 2.1. Materials

HES having average molecular weight (M_w_) of 20 kDa and a molar hydroxyethyl substitution degree of 0.5 was procured from Wuhan HUST Life Science & Technology Co., Ltd. (Wuhan, China). Indocyanine green (ICG) and Tween-80 (98%) were purchased from Aladdin Inc. (Shanghai, China). Doxorubicin (DOX) was bought from Beijing Huafenglianbo Technology Co., Ltd. (Beijing, China). All other chemicals were of analytical grade and purchased from Sinopharm, China. Reagents and solvents were used as received except for dimethyl sulfoxide (DMSO) that was purified by distillation and dried with 4 Å molecular sieves before use.

### 2.2. Preparation of HES-SS-DOX@ICG NPs

A HES-SS-DOX conjugate with a DOX load of 7.63% was first synthesized following the methods described in our previous study [9]. HES-SS-DOX@ICG NPs was prepared by directly mixing the HES-SS-DOX conjugates and ICG together. A HES-SS-DOX conjugate aqueous solution with a concentration of 10 mg/mL was first prepared. This solution was then added with varied amounts of ICG to produce a series of HES-SS-DOX/ICG solutions that had their molar ratios of ICG to DOX in the range between 1:1 and 1:4. These solutions were stirred at room temperature for at least 12 h. They were then dialyzed against deionized water for 24 h using membrane tubes (MW Cutoff: 3.5 k) to remove unbound ICG, and lyophilized to achieve HES-SS-DOX@ICG NPs. Current and subsequent ICG-involved experiments were all conducted in the dark. The optimized HES-SS-DOX@ICG NPs with the weight ratio of DOX to ICG at around 2.3 were used for the follow-up experiments, and relevant parameters for them are summarized in Table 1.

### 2.3. Characterization of HES-SS-DOX@ICG NPs

The morphology of HES-SS-DOX@ICG NPs was viewed using a transmission electron microscope (TEM JEM-100XII). Samples for TEM examinations were prepared by depositing HES-SS-DOX@ICG NP suspensions onto a carbon-coated copper grid, staining with 2% phosphotungstic acid and air-drying at room temperature. The size of more than 200 NPs in their TEM images was measured in a random manner and their average size was calculated. Zeta (ζ) potential of NPs was determined by the dynamic light scattering method (Zetasizer ZEN3690, Malvern, UK) using ultrapure water as the dispersant. Fluorescence spectra of NPs were recorded on a fluorescence spectrometer (F-4500, Hitachi, Tokyo, Japan) to monitor the fluorescence intensity of ICG under conditions of λ_ex_ = 768 nm and λ_em_ = 820 nm. The amount of DOX and ICG in HES-SS-DOX@ICG NPs was determined by using an ultraviolet spectrophotometer at 490 nm and 780 nm, respectively. Drug loading (DL) of NPs was calculated using the following formula.

(1) $$\mathrm{DL}\left(\%\right)=\frac{W_{d}\left(\mathrm{drug}\right)}{W_{n}\left(\mathrm{nanoparticles}\right)}\times 100\%$$

where W_d_ is the weight of DOX or ICG in the NPs, and W_n_ is the weight of the NPs.

### 2.4. Stability Assessment

A HES-SS-DOX@ICG solution with its equivalent DOX concentration of 0.05 mg/mL and the ratio of DOX to ICG at ca. 2.3 was prepared by dispersing HES-SS-DOX@ICG NPs in PBS (pH 7.4). A free DOX/ICG blend solution with the same DOX concentration and the same DOX/ICG ratio was also prepared by dissolving free DOX and free ICG in PBS (pH 7.4). Furthermore, 3 mL of each solution was stored at ambient temperature, and photographs for these two types of solutions were taken at various time intervals. In addition, these solutions with a volume of 10 mL for each were stored at 37 °C and their ICG stability was assessed with an ultraviolet spectrophotometer by withdrawing 2 mL of solution each time at predetermined time points (0, 2, 6, 10, and 14 days).

### 2.5. In Vitro Photothermal Effect Assessment

HES-SS-DOX@ICG NPs were dispersed in deionized water to prepare three kinds of solutions with varied ICG concentrations at 5, 10, and 20 μg/mL, and, concomitantly, having their DOX concentrations at 11.5, 23, and 46 μg/mL, respectively. In addition, a free ICG solution with its ICG concentration of 10 μg/mL was also prepared for making comparisons. In addition, 1 mL of each solution was taken and introduced into the corresponding glass vial. These vials were then irradiated by a 0.75 W/cm^2^ laser irradiation (λ = 808 nm) for 5 min while being photographed with an infrared thermal imager (Ti32, Fluke, Drexel Hill, PA, USA) every 30 s counting from the starting point of the laser irradiation. Deionized water and an HES-SS-DOX solution in deionized water with its equivalent DOX concentration at 23 μg/mL were used as controls.

### 2.6. In Vitro Drug Release

Release patterns of DOX from HES-SS-DOX@ICG NPs were determined using a dialysis method. In brief, 1 mL of HES-SS-DOX@ICG solution in deionized water (equivalent DOX amount: 40 μg, weight ratio of DOX to ICG: 2.3) was introduced into a dialysis tube (MW cutoff, 3.5 k), and the sealed tube was submerged in 30 mL of PBS (pH = 7.4) containing 0.5% Tween 80. Dithiothreitol (DTT), which is a prevailing analog of L-Glutathione (GSH), was employed as a reducing agent to assess the redox-responsive property of the HES-SS-DOX@ICG NPs since it is able to easily cleave the disulfide bonds bridging between HES and DOX. Four groups (*n* = 3) of release experiments were conducted in 4 different situations depending on whether or not DTT and laser irradiation were applied: control (saline), low DTT dose (2 μmol/mL), high DTT dose (2 mmol/mL), and high DTT dose together with laser irradiation (DTT, 2 mmol/mL, laser irradiation, 0.75 W/cm² for 5 min). The release system was maintained at 37 °C with shaking at a speed of 150 rpm. At predetermined time points, 1 mL of release medium was withdrawn while replenishing with the same volume of free buffer, and cumulative release of DOX was determined by an ultraviolet spectrophotometer (λ_ex_ = 503 nm and λ_em_ = 554 nm).

### 2.7. Cell Lines and Animals

Murine hepatoma H22 cell line and human liver hepatocellular HepG2 cell line were purchased from the Type Culture Collection of the Chinese Academy of Sciences (Shanghai, China). They were, respectively, expended in RPMI-1640 and DMEM, supplemented with 10% fetal bovine serum, 1% penicillin, and 1% streptomycin at 37 °C in a 5% CO_2_ atmosphere. These two types of cells were then resuspended in PBS for further use.

Male BALB/c mice (5 to 6 weeks old) were purchased from the Hubei Province Center for Disease Control and Prevention (Wuhan, China). The animals were housed in an air-conditioned atmosphere with relative humidity of 50% under natural light/dark cycle conditions and allowed free access to standard food and water. All animal experiments were approved by the Animal Care and Use Committee of Huazhong University of Science and Technology.

### 2.8. In Vitro Cellular Uptake

HepG2 cells were seeded into different confocal cover glasses (10^5^ cells/glass) and filled with 1.0 mL of medium. After 24 h of culturing, cell groups were exposed to their respective media containing free DOX (19.30 μg/mL), free ICG (8.13 μg/mL), and HES-SS-DOX@ICG NPs (19.30 μg/mL DOX and 8.13 μg/mL of ICG), respectively. After incubation for an additional 4 h, cells in different groups were washed thrice with PBS and fixed with a 4% paraformaldehyde solution for 20 min prior to the confocal imaging (Leica TCS SP5, Germany, λ_em_ = 488 nm for DOX and λ_em_ = 633 nm for ICG).

### 2.9. MTT Assay

HepG2 cells were used to assess the cytotoxicity mediated by different chemo-photothermal treatments. HepG2 cells were seeded in wells of 96-well culture plates at a density of 5 × 10^4^ cells/well with complete medium for 24 h. Subsequently, cell groups were treated with free DOX, free ICG, free DOX/ICG blend, and HES-SS-DOX@ICG NPs for 24 h, respectively. The amount of DOX and ICG in the applied four kinds of solutions was altered at the same pace and the ratio of DOX to ICG was maintained at 2.3. Several such-treated cell groups were further exposed to a laser at an irradiation dose of 0.75 W/cm^2^ for 5 min. The cell viability for cell groups with or without laser irradiation treatments was assessed using an MTT assay. The cell group without any mentioned treatments was used as a control, designating as 100% cell viability.

### 2.10. In Vivo Imaging and Biodistribution

The hepatic carcinoma model was established by subcutaneous injection of 2 × 10^6^ H22 cells into the right lower back of BALB/c mice (18–22 g). H22-tumor-bearing mice were randomly assigned into two groups with three mice in each group. Mice in group 1 were intravenously injected with 0.1 mL of HES-SS-DOX@ICG solution containing an equivalent ICG concentration of 30 μg/mL (ratio of DOX to ICG: 2.3). Mice in group 2 were administered with 0.1 mL of 30 μg/mL free ICG solution. Whole-body fluorescence images of mice were recorded on a near infrared (NIR) fluorescence imaging instrument (Caliper IVIS Lumina II, λ_ex_ = 740 nm, λ_em_ = 780–820 nm, PekinElmer, Santa Clara, CA, USA) and images were taken starting at 1 h after post-injection. During the imaging period, a small amount of 3% isoflurane anesthesia was intermittently applied to mice using a nose cone tube for continuous anesthesia when they were exposed to the camera. At the end of 24-h imaging, all of the mice were sacrificed by cervical dislocation. Tumors as well as major organs such as heart, liver, spleen, lung, and kidney were excised for ex vivo imaging using the same NIR fluorescence instrument.

### 2.11. In Vivo Photothermal Effect Assessment

H22-tumor-bearing BALB/c mice were randomly divided into 4 groups (*n* = 3) when the tumor volume reached about 180 mm^3^. Mice in these groups were administered with PBS, free ICG, free ICG/DOX blend, and HES-SS-DOX@ICG NPs, respectively, via the tail vein injection. The applied equivalent amount of DOX and ICG for the involved groups was 8 mg/kg and 3.4 mg/kg, respectively. At 1 h after injection, mice were anaesthetized and their tumor areas were irradiated by laser (0.75 W/cm^2^, λ = 808 nm) for 5 min. Temperature changes in the tumor areas were monitored by using a NIR thermal imager (Ti32, Fluke, Drexel Hill, PA, USA) and data were recorded starting from the beginning of the laser irradiation at a step-size of 30 s during the whole laser irradiation period. At 1 h post-irradiation, tumors were excised from the treated mice, fixed with a 4% parafomaldehyde solution, and sectioned into slices (5 μm in thickness) for subsequent histopathological analysis using hematoxylin and eosin (H/E) staining.

### 2.12. In Vivo Anti-Cancer Efficiency

H22-tumor-bearing mice were randomly divided into 10 groups (*n* = 4) based on the assigned treatment: (1) control (PBS), (2) PBS + laser irradiation, (3) free ICG,; (4) free ICG + laser irradiation, (5) free DOX, (6) free DOX + laser irradiation, (7) free DOX + free ICG, (8) free DOX + free ICG + laser irradiation, (9) HES-SS-DOX@ICG NPs, and (10) HES-SS-DOX@ICG NPs + laser irradiation. The applied equivalent amount of DOX and ICG was 8 mg/kg and 3.4 mg/kg, respectively, for the groups that were administered with DOX and/or ICG. Intravenous administration was started when the tumor volumes reached the range between 80 and 100 mm^3^. The body weight of mice was measured every two days, and the tumor volume was estimated using the following formula:

(2) $$\mathrm{Tumor}\;\mathrm{volume}=\left(\mathrm{Length}\times {\mathrm{Width}}^{2}\right)/2$$

At the end of the 14-day treatment, mice were sacrificed and residual tumors were excised for their weight determination and the relevant analysis. Additionally, major organs, including the heart, the liver, the spleen, the lungs, and the kidneys, were also harvested, fixed with a parafomaldehyde solution, and sectioned into slices (5 μm in thickness) for subsequent histopathological analysis using hematoxylin and eosin (H/E) staining. The H/E stained sections were viewed by a pathologist. Injection for all mice was conducted only once and the mice were exposed to the laser irradiation (0.75 W/cm^2^, λ = 808 nm, 5 min) once at 1 h after injection. The day for conducting the injection and irradiation was designated as day 0.

### 2.13. Statistical Analysis

Data were presented as mean ± standard deviation. Student’s *t*-test and one-way analysis of variance were performed to determine the differences in different groups, respectively. Differences were considered be statistically significant at a level of *p* < 0.05.

## 3. Results and Discussion

### 3.1. Characterization of HES-SS-DOX@ICG NPs

HES-SS-DOX@ICG NPs were prepared using a facile but highly effective method based on the mutual interactions between DOX and ICG. A series of HES-SS-DOX@ICG NPs was prepared to screen out appropriate parameters for them. HES-SS-DOX@ICG NPs with a weight ratio of DOX to ICG at about 2.3 and an average size of around 170 nm were found to have the optimum properties against tumors, and, hence, they were used for all NP-involved experiments in this study.

Photographs in Figure S1B provide visual evidence for the occurrence of interactions between free DOX and free ICG. In this study, the free DOX/ICG solution was prepared by dissolving DOX and ICG together in PBS with stirring. It was found that free DOX/ICG solution became somewhat flocculent after 6 h of stirring. Some floccus in the solution began to precipitate after about 12 h of stirring and most of the floccus were already precipitated to the bottom of the vial after stirring for 24 h (see white arrows in Figure S1B). The change in color of the free DOX/ICG solution and the formation of dark brown precipitates confirm that ICG has been bound to DOX since both the free DOX solution and the free ICG solution with the designated concentration are completely transparent, and have their own colors (Figure S1A). Some changes in colors were also observed when preparing the HES-SS-DOX@ICG solution. As shown in Figure S1A, the freshly prepared HES-SS-DOX solution in PBS showed an aurantiacus color while the free ICG solution in PBS had a pale green color. After adding HES-SS-DOX conjugates and ICG into PBS at the formulated ratio and blending them, the color of the resulting HES-SS-DOX@ICG solution turned dark brown rapidly, and no precipitate was observed during the 24-h stirring process (Figure S1C). The color change suggests that ICG molecules have been bound to HES-SS-DOX conjugates since the colors originally belonged to HES-SS-DOX conjugates and ICG have already disappeared, respectively. Considering the fact that HES-SS-DOX conjugates themselves are a kind of NPs [9], the ICG-bound HES-SS-DOX conjugates should, thus, be present in the solution in the form of HES-SS-DOX@ICG NPs. The photographs in Figure S1C exhibit that these HES-SS-DOX@ICG NPs were well dispersed in their solution without formation of any precipitate during the various stirring durations. The good dispersion of HES-SS-DOX@ICG NPs in these solutions could be ascribed to the contribution of their HES component. Since HES and the synthesized HES-SS-DOX conjugates are all fully water-soluble and the employed HES has its molecular weight of ca. 20 kDa, which is much higher than that of free DOX or free ICG, it can, thus, be envisioned that HES-SS-DOX conjugates would provide a large enough buoyant force to carry the bound ICG without inducing the formation of precipitates.

**Figure 1:**
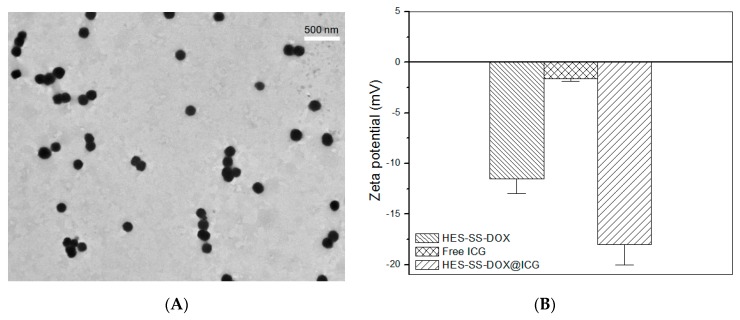

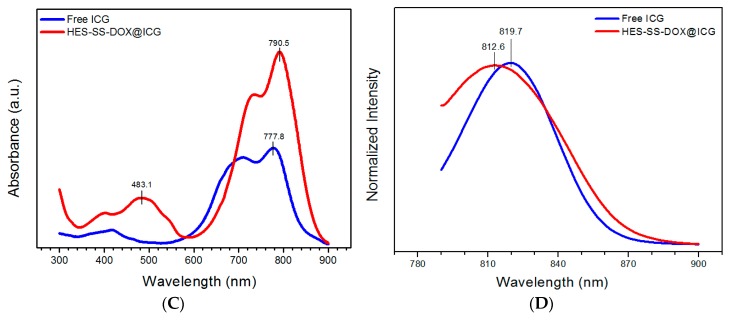
Typical TEM image (**A**) of HES-SS-DOX@ICG NPs, ζ potential, (**B**) UV absorbance spectra, (**C**) and fluorescence emission spectra (**D**) for different samples.

Previous investigations into the branched HES revealed that it had a notably higher ability to resist the α-amylase-mediated degradation as compared to linear HES [24,25], and, hence, in the present instance, the branched HES was selected as a starting material for the synthesis of HES-SS-DOX conjugates in order to endow HES-SS-DOX@ICG NPs with a prolonged circulation lifetime. Several studies have pointed out that the branched HES molecules and the resulting HES-SS-DOX conjugates in their hydrated state are in the rough shape of microspheres with the size of about 10 nm or somewhat larger [9,24,25]. It can, thus, be conjectured that HES-SS-DOX@ICG NPs would have a similar size because ICG is a kind of small molecule and its content in HES-SS-DOX@ICG NPs is also low. This conjecture is unfortunately inconsistent with the actual observation. A typical TEM image for HES-SS-DOX@ICG NPs shows that they had their size range between about 100 and 200 nm (Figure 1A), which is much larger than the size of HES-SS-DOX conjugates. This implies that these HES-SS-DOX@ICG NPs should be aggregates formed by a number of ICG-carried HES-SS-DOX conjugates.

It is know that ICG has highly conjugated electron configuration while showing an amphiphilic characteristic due to the presence of two polycyclic indole skeletons and two sulfate groups in its molecular structure [23,26]. A previous study reported that ICG was able to collaboratively assemble with epirubicin (EPI) to form ICG-EPI NPs via electrostatic, π–π stacking, and hydrophobic interactions. The resulting ICG-EPI NPs have their size of around 200 nm with EPI loading as high as ca. 92% [23]. The results for ICG-EPI NPs suggest that (1) an ICG molecule can interact with several EPI molecules to form partners, and vice versa. The results also suggest that (2) the total binding force generated by different interactions between ICG molecules and EPI molecules can only bind a limited number of ICG and EPI molecules to form structurally stable aggregates, and the size of ICG-EPI NPs depends on the ratio of ICG to EPI. Taking into consideration the fact that DOX is extremely similar to EPI in structure, ICG molecules should also be able to interact with DOX molecules in the same way. In the present situation, ICG molecules are required to interact with HES-SS-DOX conjugates because DOX is already loaded onto HES. Since HES-SS-DOX conjugates are nearly spherical in shape [9], some ICG molecules could be bound to the DOX molecules located inside HES-SS-DOX conjugates whereas others would be bound to the DOX molecules that reside in the superficial regions of HES-SS-DOX conjugates. As a result, only those ICG/DOX partners in the superficial regions of different HES-SS-DOX conjugates can further interact with each other and provide a collaborative force to bind a certain number of HES-SS-DOX conjugates together to aggregate into structurally stable HES-SS-DOX@ICG NPs, as observed in Figure 1A.

The size of drug-loaded NPs is known to play a key role in regulating the in vivo performance of the NPs. It has been suggested that NPs with a size less than 200 nm are likely to accumulate in the tumor via the EPR effect, and large NPs such as those larger than 300 nm would be easily caught by RES in the liver and spleen [1,27,28]. Therefore, the size of HES-SS-DOX@ICG NPs needs to be well modulated. In this study, besides the size of HES-SS-DOX@ICG NPs, the ratio of DOX to ICG is also an important parameter for their anti-cancer efficiency. The size and composition of HES-SS-DOX@ICG NPs were synchronously optimized by mainly altering the molar ratio of HES-SS-DOX conjugates and ICG. Since the absorption wavelength of HES-SS-DOX@ICG NPs partially overlaps with the detection wavelength of the bound ICG inside NPs, the size of HES-SS-DOX@ICG NPs was determined using their TEM images rather than the regularly used dynamic light scattering instrument. Based on TEM image analyses, the mean size of HES-SS-DOX@ICG NPs was measured as ca. 170 nm, which signifies that they are suitable for in vivo usage from the perspective of the size account. ζ potential of ICG, HES-SS-DOX, and HES-SS-DOX@ICG NPs was also measured, and data are depicted in Figure 1B. It can be seen that ICG and HES-SS-DOX conjugates were negatively charged with ζ potential of around 1.6 and 11 mV and HES-SS-DOX@ICG NPs had their ζ potential of about 18 mV, which is significantly greater (*p* < 0.05) than the sum of ζ potentials of ICG and HES-SS-DOX conjugates. This demonstrates that charges in the HES-SS-DOX@ICG NPs have been redistributed due to the collaborative interactions occurring between ICG and HES-SS-DOX conjugates.

The UV absorbance and fluorescence emission spectra of ICG and HES-SS-DOX@ICG NPs were detected and they are represented in Figure 1C,D, respectively. Curves in Figure 1C explicate that free ICG had a wide absorption band with the maximum intensity at about 777 nm.

With respect to HES-SS-DOX@ICG NPs, the band at ca. 483 nm was ascribed to their DOX component and the wide band with its shape that is somewhat similar to that for free ICG was moved to the higher wavelength interval, which is characterized by the maximum intensity at ca. 790 nm. In comparison to free ICG, the wavelength at the maximum intensity for HES-SS-DOX@ICG NPs was red-shifted by about 13 nm. In Figure 1D, a pronounced difference in wavelength was also detected when the maximum fluorescence intensity for free ICG was compared with that for HES-SS-DOX@ICG NPs. On the basis of these results, it can be inferred that the collaborative force arisen from electrostatic, hydrophobic, and π–π stacking interactions between ICG molecules and HES-SS-DOX conjugates has driven them to organize into certain assemblies, which leads to significant wavelength shifts occurring for the ICG component in HES-SS-DOX@ICG NPs. The similar wavelength shifts for ICG have also been mentioned in some studies where ICG and DOX were used together for chemo/photothermal combination therapies, and our findings are basically consistent with the reported results [23,29,30]. 

HES-SS-DOX@ICG NPs were already optimized to achieve high therapeutic efficiency and minimize the side effects of DOX, and the drug load of DOX and ICG in HES-SS-DOX@ICG NPs was formulated as 7.7% and 3.2%, respectively. Although a higher ICG amount could be loaded into HES-SS-DOX@ICG NPs, it was found that such formulated HES-SS-DOX@ICG NPs were able to fulfill the mission for the tumor eradication in vivo. Several major parameters for the optimal HES-SS-DOX@ICG NPs are summarized in Table 1.

### 3.2. Stability Assessment

The physical stability of NPs is known to be closely associated with their performance [27,28]. HES-SS-DOX@ICG NP solutions in PBS were, thus, stored at ambient temperature for various durations up to seven days to test if any changes happened to them. Photographs in Figure S2 exhibited that these solutions remained transparent without changes in their colors or formation of any precipitates during seven-day storage, which verifies that they are stable in PBS.

**Figure 2:**
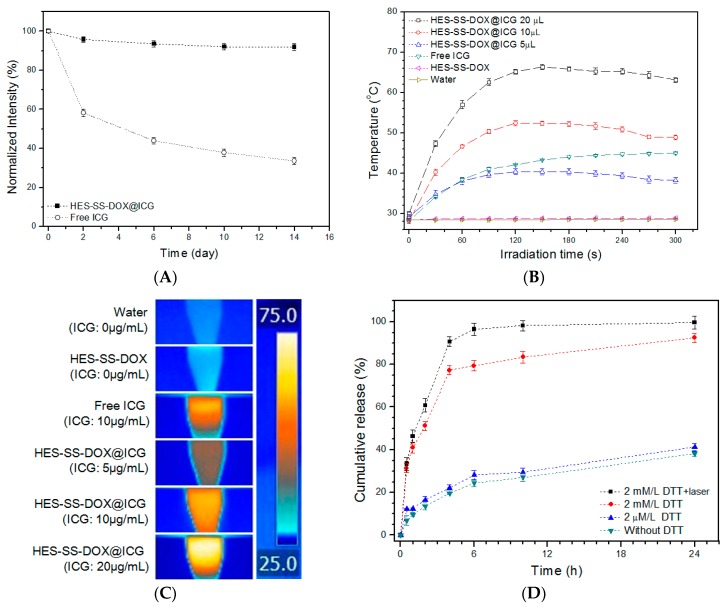
UV absorbance intensity (**A**, in PBS) detected at different time points after preparation of the stock solutions. Photothermal profiles (**B**, in deionized water, concentration of free ICG solution: 10 μg/mL) of different samples, infrared themographic images (**C**, photos were taken at the moment after 5-min irradiation) of different samples, and cumulative release (**D**) of DOX from HES-SS-DOX@ICG NPs at different DTT concentrations but at a fixed pH of 7.4 with or without laser irradiation (irradiation intensity and time for all involved cases were 0.75 W/cm^2^ and 5 min. The ratio of DOX to ICG for the related cases was 2.3).

ICG has been used as imaging or photothermal agents for diagnostic and therapeutic purposes [15,16]. Despite its many applications, ICG usually shows poor stability in aqueous media and is prone to aggregate. The former could lead to short-lived ICG and the latter would cause the low efficiency of ICG. HES-SS-DOX@ICG NPs were tested to assess whether they have an ability to maintain the stability of the loaded ICG, and relevant results are presented in Figure 2A. After two-day storage, the intensity of HES-SS-DOX@ICG NPs was remained at around 96% but the intensity of free ICG already dropped to 58% of its initial value. Thereafter, the UV absorbance intensity of free ICG progressively decreased until it reached approximately 34% at the end of two weeks. On the other hand, the absorbance intensity of HES-SS-DOX@ICG NPs decreased only slightly within two weeks and it remained as high as 92% on day 14. These results demonstrate that HES-SS-DOX@ICG NPs can effectively maintain the stability of the loaded ICG in aqueous medium. The protective effect of HES-SS-DOX@ICG NPs on the loaded ICG molecules can be attributed to the composition and structure of NPs themselves. As mentioned early, HES-SS-DOX@ICG NPs were composed of a number of ICG-bound HES-SS-DOX conjugates, and, hence, most ICG molecules were encapsulated inside the HES-SS-DOX@ICG NPs. Accordingly, in the HES-SS-DOX@ICG solution, most ICG molecules inside HES-SS-DOX@ICG NPs would not be directly exposed to the aqueous environment, which leads to their high stability.

### 3.3. Assessment of In Vitro Photothermal Effect

The photothermal conversion efficiency of HES-SS-DOX@ICG NPs was monitored to evaluate whether these NPs are able to preserve the deserved efficiency of the loaded ICG. In this study, the time and intensity of laser irradiation were set as 5 min and 0.75 W/cm^2^, respectively, since several studies did for measuring the photo-thermal effect of ICG-involved agents. Figure 2B presents temperature profiles for different samples that were exposed to the laser irradiation for 5 min. Three kinds of HES-SS-DOX@ICG solutions showed elevating temperatures and their maximum temperature (T_max_), which corresponds to the equilibrium temperature between the ICG-induced heat production and the heat loss of solutions, reached about 40 °C, 52 °C, and 66 °C after being irradiated for around 150 s, respectively. Afterward, the temperatures matching with HES-SS-DOX@ICG solutions slightly dropped. In addition, it is also observed that, as the ICG amount in HES-SS-DOX@ICG solutions increased from 5 to 20 μg/mL, the temperature curve moved toward the high temperature region in an ICG-dose dependent manner. Similar temperature responsive patterns were also commonly observed in other ICG-involved photothermal therapeutic systems [17,20]. The reason for the small temperature drops after T_max_ could be that the ICG-induced heat production is slightly less than the heat loss caused by the environment because T_max_ for these HES-SS-DOX@ICG solutions is significantly higher than the ambient temperature. In the case of free ICG solution (10 μg/mL), it had a notably slower temperature rising rate during the irradiation when compared with its HES-SS-DOX@ICG 10 μg/mL partner, and its T_max_ reached about 44 °C after irradiation for ca. 5 min, which was significantly lower than that (49 °C) for its HES-SS-DOX@ICG 10 μg/mL partner. With regard to two control samples including the HES-SS-DOX solution and water, their temperature was almost equal to their initial temperature after 5 min of irradiation without measurable changes. Figure 2C provides a group of images taken from different samples that were exposed to irradiation for 5 min. Visual differences in colors can be viewed from these samples. Results presented in Figure 2A–C verify that HES-SS-DOX@ICG NPs can effectively preserve the stability and photothermal efficiency of the loaded ICG. Based on these results, all subsequent experiments involved in photothermal effects were conducted under the same irradiation conditions (5 min, 0.75 W/cm^2^).

### 3.4. In Vitro Drug Release

To determine the release patterns of HES-SS-DOX@ICG NPs, HES-SS-DOX@ICG solutions in deionized water were dialyzed against different buffer solutions with or without DTT as an analog of GSH at 37 °C for 24 h. As shown in Figure 2D, HES-SS-DOX@ICG NPs behaved in a DTT-dose dependent fashion. The DOX amount released from HES-SS-DOX@ICG NPs sharply increased when the applied DTT dose was changed from 2 μM/mL to 2 mM/mL. After being exposed to the medium containing 2 mM/mL of DTT over 24 h, HES-SS-DOX@ICG NPs released about 92% of the loaded DOX. On the other hand, these NPs were only able to release around 38% of the loaded DOX during the same period of sampling time if they were in a medium without containing any DTT. The results clearly reveal that HES-SS-DOX@ICG NPs have high sensitivity to DTT, which is a widely used analog for GSH. Figure 2D also shows that the DOX release from HES-SS-DOX@ICG NPs would become faster if these NPs were exposed to the medium containing 2 mM/mL of DTT together with laser irradiation when comparing the case without irradiation, which suggests that the release patterns of HES-SS-DOX@ICG NPs can be cooperatively regulated by the reducing agent and the applied laser irradiation. Such a redox-responsive release features of HES-SS-DOX@ICG NPs will be highly propitious to their in vivo applications. Based on these release patterns, it can be inferred that once HES-SS-DOX@ICG NPs arrive at the tumor sites via passive EPR effect, the applied laser irradiation would stimulate the ICG-induced heat production for the destruction of the tumor matrix. After being further internalized by cancer cells, the HES-SS-DOX@ICG NPs would fast release the loaded DOX due to the double trigger arisen from the high level of intracellular GSH and the applied irradiation.

### 3.5. In Vitro Cellular Uptake

**Figure 3:**
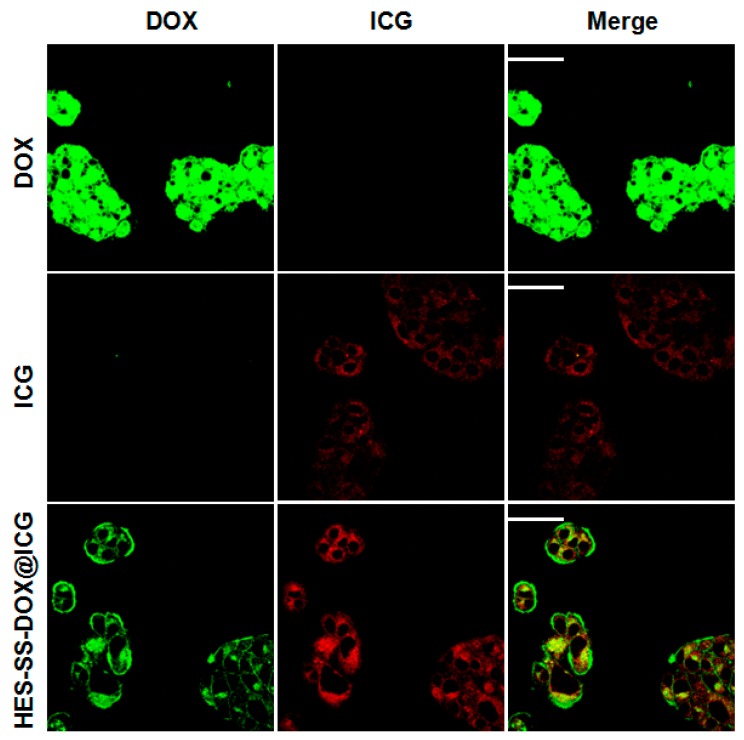
Subcellular localization of free DOX, free ICG, and HES-SS-DOX@ICG NPs in HepG2 cells after 4 h of co-incubation (scale bar: 50 μm).

The subcellular localization of free DOX, free ICG, and HES-SS-DOX@ICG NPs in HepG2 cells was investigated and the results are represented in Figure 3. After 4 h of incubation, free DOX was found to mainly localize inside the cell nucleus of HepG2 cells, which is consistent with our previous results [9]. With respect to free ICG, the image shows that it was homogenously distributed in the cytoplasm of HepG2 cells, which is similar to the observation in previous studies where ICG was found to mainly bind to intracellular proteins (glutathione S-transferase) [20,31]. As for HES-SS-DOX@ICG NPs, the matched image exhibits that DOX was located in both the cytosol and the nucleus of HepG2 cells. The possible reason is that some DOX molecules released from the internalized HES-SS-DOX@ICG NPs further entered into the cell nucleus. However, some other DOX molecules were still trapped inside HES-SS-DOX@ICG NPs because the co-incubation was 4 h and HES-SS-DOX@ICG NPs need a longer time for internalization as well as the following DOX release.

### 3.6. MTT Assay

**Figure 4:**
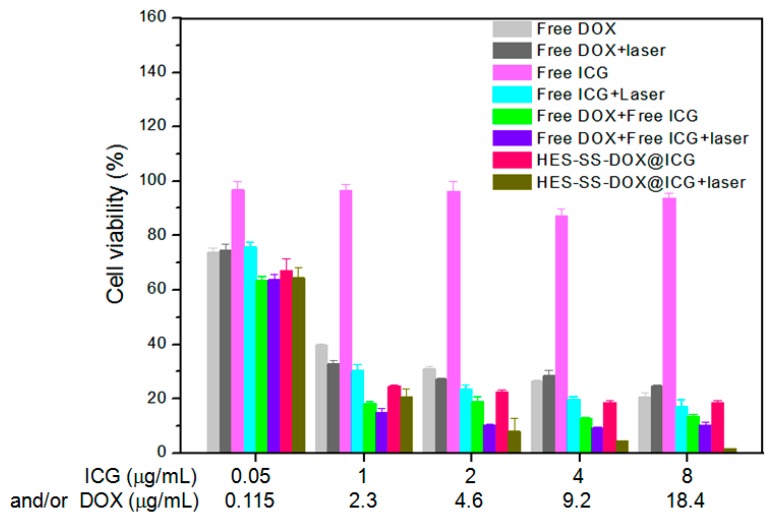
Viability versus ICG concentration for HepG2 cells treated with free DOX, free ICG, free DOX + free ICG, and HES-SS-DOX@ICG NPs with or without laser irradiation (culture time: 24 h, irradiation intensity, and time for all involved cases were 0.75 W/cm^2^ and 5 min).

Effects of free DOX, free ICG, HES-SS-DOX, or HES-SS-DOX@ICG NPs on the viability of HepG2 cells were evaluated and the results are illustrated in Figure 4. The cell viability in free ICG group without irradiation treatment was remained at about 90% or higher when the ICG concentration was altered from 0.05 to 8 μg/mL, which indicates that free ICG is nearly nontoxic to these cells. In other groups, cell viability significantly decreased due to the cytotoxicity stemmed from DOX, or irradiation-excited ICG, or a combination of both. Since the equivalent ratio of DOX to ICG was maintained at around 2.3, cell viability in these groups can be correlated to the amount of either ICG or DOX. Except for free ICG group with no irradiation treatment, cell activity in other groups showed downward trends as the applied amount of DOX or ICG increased. In comparison to other groups, HES-SS-DOX@ICG NPs together with irradiation treatment had significantly higher cytotoxicity to HepG2 cells when the applied ICG dose was 2 μg/mL or higher. In particular, the cells that were treated with HES-SS-DOX@ICG NPs + laser (ICG: 8 μg/mL, DOX: 18.4 μg/mL) for 24 h were almost all dead, which demonstrates that this combinatorial treatment has the potential to fully inhibit the growth of HepG2 cells. It is quite ascertained that the treatment based on HES-SS-DOX@ICG NPs + laser has much higher anti-cancer efficiency compared to free DOX when the applied DOX amount increases from 0.115 to 18.4 μg/mL. The high anti-cancer efficiency of irradiation-excited HES-SS-DOX@ICG NPs can be attributed to the local hyperthermia arisen from the excited ICG and the fast DOX release due to the GSH-induced cleavage of disulfide bonds and the heat-assistance effect (Figure 2D). These results corroborate that the presently developed HES-SS-DOX@ICG NPs can act as a potential therapeutic agent for efficient chemo/PTT cancer treatments.

### 3.7. In Vivo Imaging and Bio-Distribution

**Figure 5:**
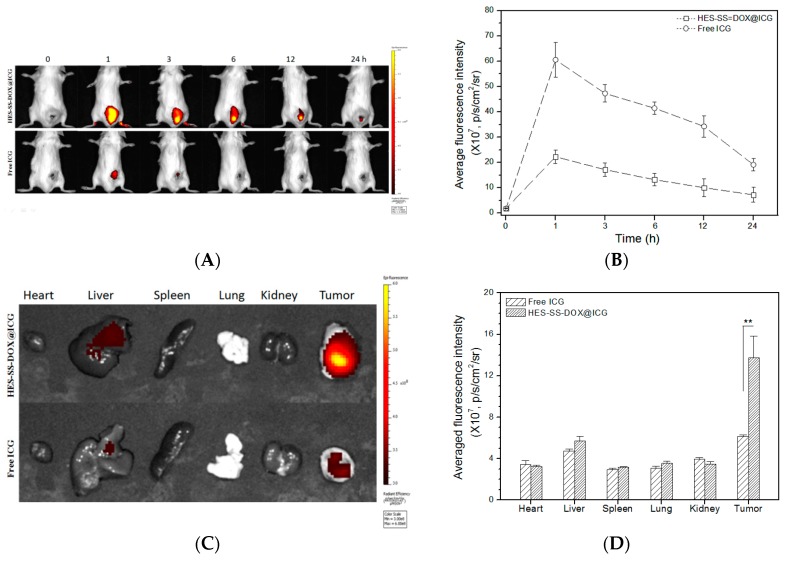
Representative fluorescence images and average fluorescence intensity. Real-time fluorescence images (**A**) of H22-tumor bearing BALB/c mice and matched fluorescence intensity (**B**) detected at different time points. (**C**) Ex vivo fluorescence images of major organs and tumors excised from two groups of mice and matched average fluorescence intensity (**D**) (harvesting time of organs and tumors in H22-tumor bearing mice, at 1 h after injection of free ICG or HES-SS-DOX@ICG NPs. ** *p* < 0.01. *n* = 4).

ICG has an intrinsic fluorescence nature, and, hence, HES-SS-DOX@ICG NPs that were accumulated in the tumor and organs can be tracked without additional radio labeling or fluorescent labeling. Figure 5A presents two sets of fluorescence images taken from the back of H22-tumor-bearing mice. In the case of free ICG group, the fluorescence signal first appeared in the tumor region after 1 h of ICG injection, and, after that, fluorescence intensity progressively decreased as time extended up to 24 h. With regard to the HES-SS-DOX@ICG NP group, their fluorescence intensity changed in a trend as similar as that for free ICG, but the magnitude of fluorescence intensity was notably higher than that for free ICG. More quantitative results for the fluorescence intensity detected from mice are presented in Figure 5B. In the HES-SS-DOX@ICG NP group, the maximum fluorescence intensity appeared at one hour post-injection and it was approximately three times as much as that of free ICG. After the peak intensity, the fluorescence intensity in the HES-SS-DOX@ICG NP group decreased but remained much higher than that in the free ICG group until the end of the detection. Figure 5A,B explicate that greatly enhanced accumulation of HES-SS-DOX@ICG NPs at the tumor site, which has occurred in comparison to free ICG. These results can be attributed to the highly hydrophilic surface properties and appropriate size of HES-SS-DOX@ICG NPs, which facilitates them to approach tumors via passive EPR effect, and results in their enhanced intra-tumoral accumulation. In contrast to HES-SS-DOX@ICG NPs, free ICG molecules could be quickly quenched or fast cleared from the body of mice due to their instability and self-aggregation features when exposed to the physiological environment. On the basis of results presented in Figure 5A,B, it can be drawn that one hour post-injection is the optimal time point to implement laser irradiation on the tumor area of mice to achieve the high photothermal effect. Figure 5C shows representative ex vivo images of major organs and tumors, and Figure 5D presents the average fluorescence intensity matched with two groups of mice. The images indicate that fluorescence signals mainly appeared in live tumors. Signals detected from liver reveal that HES-SS-DOX@ICG NPs and ICG molecules were, respectively, detained by liver to a certain extent due to the RES-rich nature of liver. Bar graphs in Figure 5D indicate that, at 24 h after injection, the average fluorescence intensity at tumors excised from the HES-SS-DOX@ICG NP group was almost twice that of free ICG group, which strongly supported that HES-SS-DOX@ICG NPs are able to protect ICG from degradation and clearance while effectively delivering both DOX and ICG toward tumors.

### 3.8. Assessment of In Vivo Temperature Profiles and Photo-Thermal Effects

**Figure 6:**
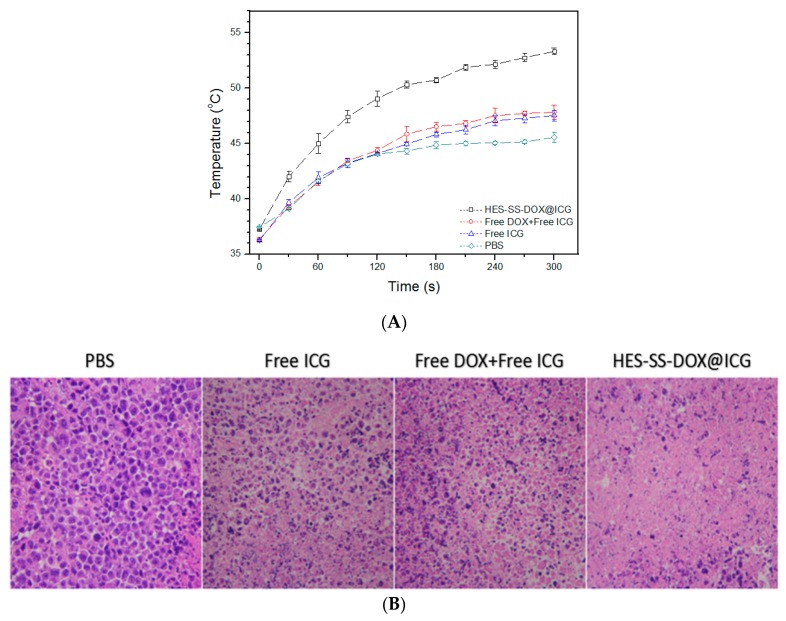
Temperature profiles (**A**) of tumor area of H22-tumor bearing mice treated with different agents for 1 h and subsequent irradiation for 5 min starting at 1 h after injection and optical micrographs (**B**) of H/E-stained sections for tumors that were excised from the treated mice at one hour post-irradiation (irradiation intensity and time for all involved cases were 0.75 W/cm^2^ and 5 min. *n* = 3, magnification, 200×).

Several formulations were tested on H22-tumor-bearing mice to assess temperature changes in the tumor area of mice that were administered with different formulations for 1 h and subsequent laser irradiation for 5 min starting at 1 h after injection. Relevant results are represented in Figure 6A. In PBS, free ICG and free ICG + free DOX groups, the endpoint temperature in the tumor area of the treated mice was detected as around 45.6 °C, 47.5 °C, and 47.8 °C, respectively, which indicates that these three agents have a limited capability to induce heat generation [15]. In contrast to the mentioned three agents, the temperature profile for HES-SS-DOX@ICG NPs elucidates that these NPs were capable of generating laser-triggered hyperthermia at the tumor area with the temperature reaching about 53 °C after 5 min of laser irradiation, and such a local temperature is sufficient to cause heat-induced cytotoxicity against cancer cells [15,22]. Such induced hyperthermia at the tumor sites can be attributed to the dual effects of HES-SS-DOX@ICG NPs. One is that the NPs tendentiously accumulate in tumors (Figure 5) and another one is that these NPs can protect the loaded ICG molecules from degradation during their circulation in vivo.

To evaluate the photo-thermal effect of applied agents on tumors of the treated mice, the irradiated tumors were excised from the mice at one hour post-irradiation and sectioned into slices for histological analysis using H&E staining. Mice in PBS groups were used as a control. As seen in Figure 6B, micrographs corresponding to free ICG and free ICG + free DOX groups exhibit that a certain number of cells in the tumors were already necrotic or apoptotic, which is characterized by the reduced cell number and irregularly shaped nuclei (indicated by a dark purple color). By comparing the area of the dark purple color among these micrographs, it can be estimated that the free ICG has certain cytotoxicity to cancer cells, which is similar to that resulted from free ICG in combination with free DOX. In contrast to free ICG or a combination of free ICG and free DOX, HES-SS-DOX@ICG NPs showed significantly higher cytotoxicity to cancer cells, as shown by the large purple-free area in the matched micrograph. As described in the experimental section, tumor-bearing mice in these groups were subjected to different agents for 1 h, and, subsequently, to laser irradiation on the tumor area for 5 min, and tumors were excised at one hour post-irradiation. These mean that tumors were removed from the treated mice just a little longer than two hours after the injection. During this period of time, it can be inferred that only a small amount of DOX could be accumulated in the tumor site when the DOX-involved formulations were applied. Even so, DOX can only exert a very limited effect on the cancer cells during such a short period of time because DOX needs a longer time to enter the nucleus and to take its actions. Accordingly, the observed impairments to cancer cells in Figure 6B should be mainly assigned to the photo-thermal effect of applied agents. Among these agents, the significantly higher anti-cancer photo-thermal cytotoxicity of HES-SS-DOX@ICG NPs can be attributed to their enhanced intra-tumoral accumulation (see Figure 5) and proactive effect on the loaded ICG molecules.

### 3.9. In Vivo Anti-Cancer Efficacy

**Figure 7:**
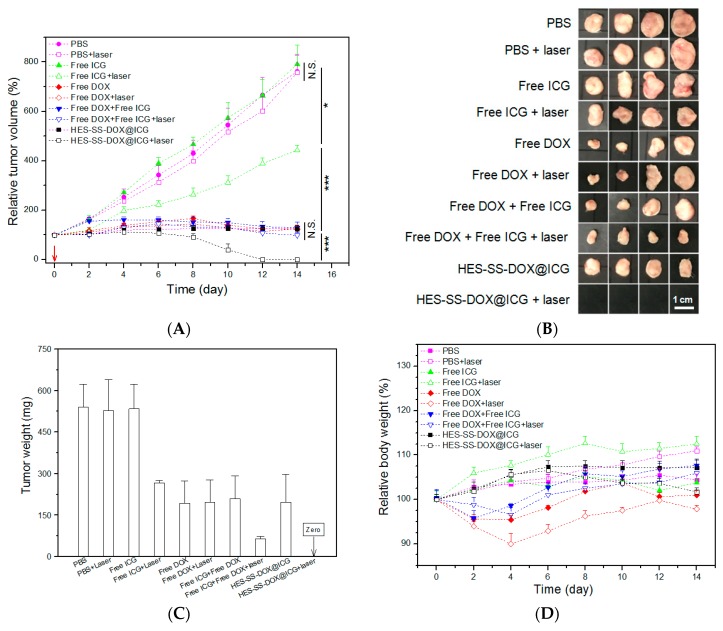
Time-dependent variations of tumor volume (**A**), images (**B**), and average weight (**C**) of tumors excised from mice after 14 days of treatment, and changes in body weight (**D**) for the treated mice (the inserted red arrow indicates the day for the injection, irradiation intensity, and time for all involved cases were 0.75 W/cm^2^ and 5 min. The applied equivalent amount of DOX and ICG was 8 mg/kg and 3.4 mg/kg. N.S., no significance. * *p* < 0.05. *** *p* < 0.001. *n* = 4).

H22-tumor-bearing mice were administered with different formulations to evaluate synergistic anti-tumor efficacy of applied agents, and results are elucidated in Figure 7A–D. As shown in Figure 7A, the tumor volume of mice in PBS, PBS + laser, and free ICG groups unremittingly increased as time advanced, which indicates that these treatments were ineffective. Chemotherapies alone basing on free DOX with or without laser irradiation, or HES-SS-DOX@ICG NPs without laser irradiation could partially inhibit the tumor growth, and treatments based on DOX with or without laser irradiation showed higher tumor growth inhibition than that corresponding to free ICG together with laser irradiation. Tumor volume versus time curves reveal that treatments associated with free DOX + free ICG, free DOX + free ICG + laser, and HES-SS-DOX@ICG NPs without laser irradiation had similar anti-tumor efficacy without significant differences among these groups. Significantly, HES-SS-DOX@ICG NPs together with laser irradiation had the highest tumor inhibition efficacy among all these groups, and all tumors in this group were fully eradicated during a 14-day treatment period within which only one injection and one single subsequent laser irradiation were performed, which was further shown by the excised tumors and the average tumor weight (Figure 7B,C). The high anti-cancer efficacy detected from the group matching with HES-SS-DOX@ICG NPs + laser irradiation can be ascribed to the synergistic effects arisen from HES-SS-DOX@ICG NPs due to their several merits, including a high photo-thermal conversion rate (Figure 2B and Figure 6A), a redox-responsive and irradiation enhanced DOX release (Figure 2D), and tendentiously intra-tumoral accumulation (Figure 5A).

In Figure 7D, weight loss was observed from four groups involving free DOX starting from around one day after the injection, which indicates toxic effects induced by free DOX. No significant weight loss was recorded for mice in six other groups during the treatment period. In particular, mice in the group corresponding to HES-SS-DOX@ICG NPs + laser irradiation had a body weight quite similar to that in the PBS group, which suggests that the toxic effects associated with free DOX can be significantly reduced given that HES-SS-DOX@ICG NPs rather than free DOX were utilized. Moreover, the treatment based on the employed HES-SS-DOX@ICG NPs is also relatively safe for combinational chemo-PTT therapy.

DOX is an anthracycline glycoside antibiotic with wide-spectrum anti-tumor activity against a range of malignancies [32,33,34,35,36]. Despite its wide use, DOX can cause severe side effects [32]. In particular, DOX-induced myocardial impairment could potentially lead to heart failure [32,35,36]. In this regard, further investigations were conducted to find out whether any cardiac impairment occurs to mice that were treated with different agents. Major organs such as heart, liver, spleen, lung, and kidney were harvested at the end of treatments and sectioned into slices for histopathological analysis. Several sets of H/E staining micrographs are presented in Figure S3. Micrographs matching with groups of free ICG and HES-SS-DOX@ICG NPs, no matter with or without laser irradiation, show that heart, liver, spleen, lung, and kidney of the treated mice had normal histological structures without visual pathological changes when comparing with the matched ones in the control group (PBS group). In DOX and DOX + ICG groups, micrographs for liver, spleen, lung, and kidney of the treated mice exhibited that these organs had normal histological structures regardless of whether laser irradiation is applied. However, the micrographs for heart showed pathological changes [37,38], as indicated by irregularly oriented arrangements of myocardial fibers and aggregated inflammatory cardiac muscle cells. Results presented in Figure 7 and Figure S3 demonstrated that presently developed HES-SS-DOX@ICG NPs have not resulted in impairments to the normal organs of the treated mice no matter whether they are used with laser irradiation.

**Figure 8:**
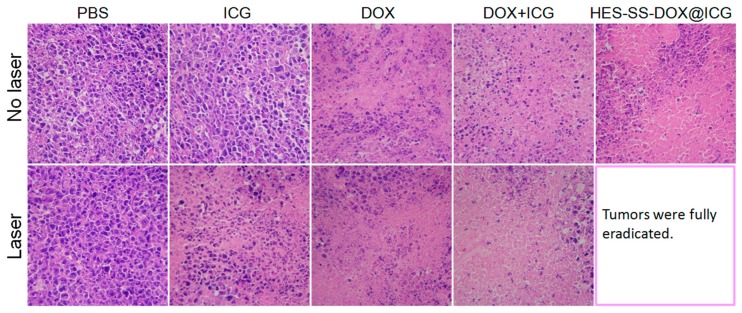
Representative optical micrographs of H/E-stained tissue sections, respectively, corresponding to tumors excised from H22-tumor-bearing mice treated with different agents (tumors were harvested after 14-day treatment, DOX equivalent, 8 mg/kg, ICG equivalent, 3.4 mg/kg, magnification, 200×).

The residual tumors harvested at the end of treatments were also sectioned into slices for histological analysis, and their H/E staining micrographs are presented in Figure 8. In comparison to the control (PBS group), many cells in the tumors of mice that were treated with free ICG + laser were necrotic or apoptotic, which is indicated by the reduced cell number and the shrunken or broken nuclei (denoted by a dark purple color). The treatments involved in free DOX with or without laser irradiation had a similar efficacy against tumors, but they seemed to be somewhat more efficient than that in the free ICG + laser group in view of the increased purple-free area in the matched micrographs. In the absence of laser irradiation, the treatment based on free ICG + free DOX only had a limited efficiency against tumors. Together with laser irradiation, the efficiency of this treatment was significantly improved, as shown by the large purple-free area in the corresponding micrograph. Similarly, only with laser irradiation, HES-SS-DOX@ICG NPs would be able to fully eradicate cancer cells. The results presented in Figure 8 were basically consistent with those elucidated in Figure 7.

## 4. Conclusions

In this study, HES-SS-DOX@ICG NPs with the designed composition and controlled size were successfully prepared. These NPs had good physical and photothermal stability in aqueous media and showed high photo-thermal efficiency when exposed to a physiological environment. They were able to quickly release the loaded DOX in response to the redox stimulus and the applied irradiation. Based on the H22-tumor-bearing mouse model, these NPs were found to tendentiously accumulate inside tumors in comparison to other major organs. HES-SS-DOX@ICG NPs together with dose-designated laser irradiation were capable of fully eradicating tumors with only one injection and one single subsequent laser irradiation on the tumor site during a 14-day treatment period, while showing almost no impairment to the body. Considering that the hydroxyethyl starch, the matrix material of the NPs, is fully biodegradable and biocompatible, and the NPs have good in vivo safety and highly efficient anti-tumor performance. The presently developed HES-SS-DOX@ICG NPs in conjugation with laser irradiation have promising potential for chemo-photo-thermal cancer therapy in the clinic.

## Figures and Tables

**Table 1:** Parameters for nanoparticles.

Sample Name	Mean Size (nm)	PDI *	DL of DOX (%)	DL of ICG (%)	ζ (mV)
HES-SS-DOX@ICG	169.1 ± 16.4	0.12	7.72 ± 0.14	3.25 ± 0.08	−18.0 ± 1.98

* PDI, polydispersity index.

## Supplementary Materials

The following are available online at http://www.mdpi.com/2072-6694/11/2/207/s1, Figure S1: Photographs for three kinds of stored solutions, Figure S2: Photographs for HES-SS-DOX@ICG solutions that were stored at an ambient temperature for various durations (dispersant: PBS, pH 7.4, equivalent DOX concentration: 0.1 mg/mL, ratio of DOX to ICG: 2.3), Figure S3: Representative micrographs of H/E-stained tissue sections, respectively, corresponding to major organs excised from H22-tumor-bearing mice treated with different agents (organs were harvested after 14-day treatment. DOX equivalent, 8 mg/kg. ICG equivalent, 3.4 mg/kg. magnification, 200×).
