# Supplementary material for: Hydroxyethyl Starch-Based Nanoparticles Featured with Redox-Sensitivity and Chemo-Photothermal Therapy for Synergized Tumor Eradication
Source: Cancers (Basel). 2019 Feb 11;11(2):207. doi: 10.3390/cancers11020207 (PMC6406889; doi:10.3390/cancers11020207)
Supplement: Supplementary file 1 [file cancers-11-00207-s001.pdf]

Supplementary Materials: Hydroxyethyl Starch-Based Nanoparticles Featured with Redox-Sensitivity and Chemo-Photothermal Therapy for Synergized Tumor Eradication

Chan Yu, Chuqi Liu, Shaocong Wang, Zheng Li, Hang Hu, Ying Wan and Xiangliang Yang

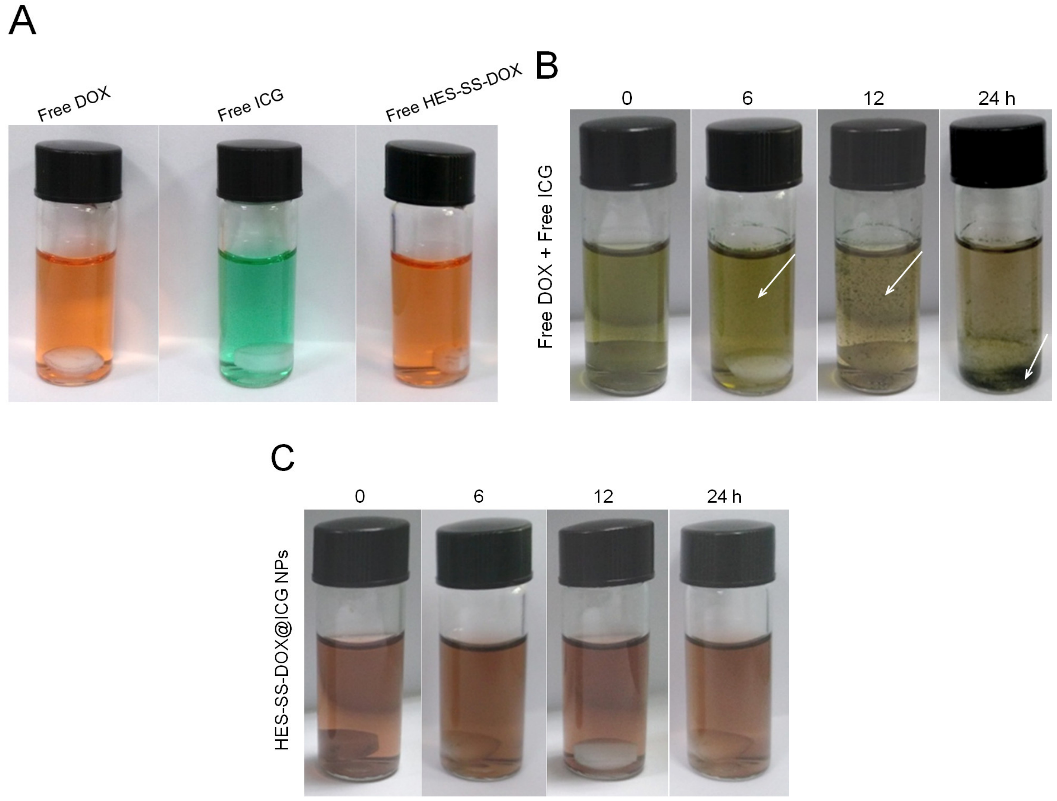

Figure S1. Photographs for three kinds of stored solutions (**A**) associated with different free solutes; for free DOX/ICG solutions (**B**) and for HES-SS-DOX@ICG solutions (**C**) (free DOX/ICG solutions or HES-SS-DOX@ICG solutions were stirred with various time intervals up to 24 h; dispersant: PBS, pH 7.4; equivalent DOX concentration: 0.1mg/mL, ratio of DOX to ICG: 2.3; three kinds of free solutions were also prepared in such a way that the ratio of DOX to ICG was 2.3 when the content of DOX was compared with the content of ICG.

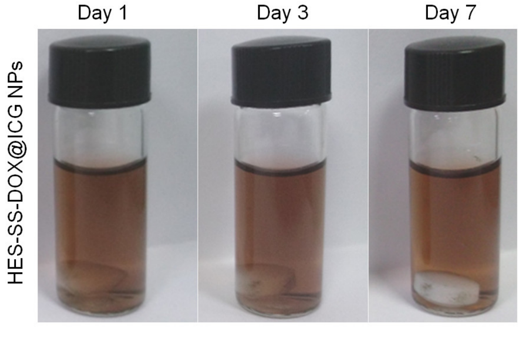

Figure S2. Photographs for HES-SS-DOX@ICG solutions that were stored at ambient temperature for various durations (dispersant: PBS, pH 7.4; equivalent DOX concentration: 0.1mg/mL, ratio of DOX to ICG: 2.3).

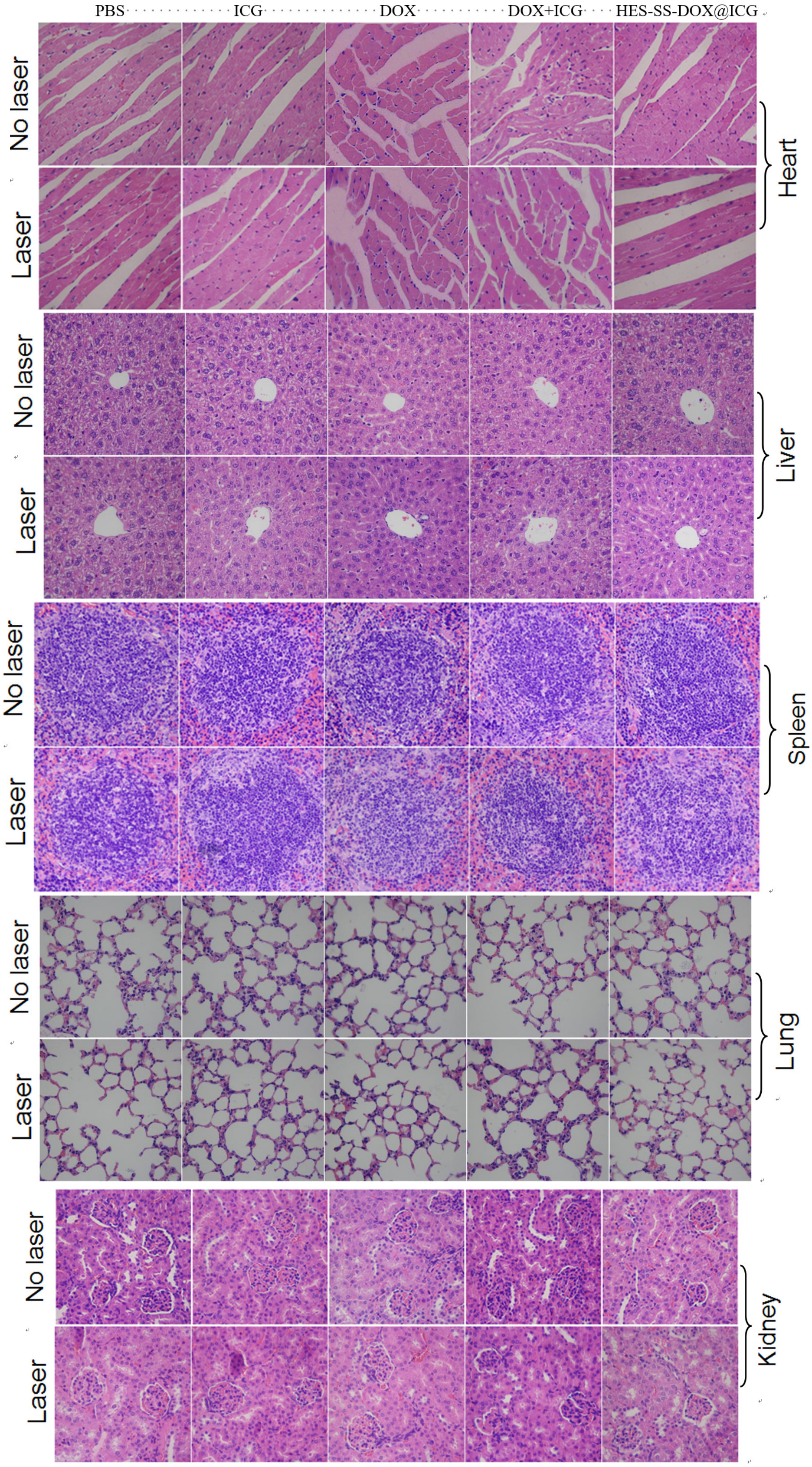

Figure 3. Representative micrographs of H/E-stained tissue sections respectively corresponding to major organs excised from H22-tumor-bearing mice treated with different agents (organs were harvested after 14-day treatment; DOX equivalent, 8 mg/kg; ICG equivalent, 3.4 mg/kg; magnification, 200×).
